# *In Silico* Screening of Mutated K-Ras Inhibitors from Malaysian *Typhonium flagelliforme* for Non-Small Cell Lung Cancer

**DOI:** 10.1155/2014/431696

**Published:** 2014-09-21

**Authors:** Ayesha Fatima, H. F. Yee

**Affiliations:** Department of Pharmaceutical Technology, Faculty of Pharmaceutical Sciences, UCSI University, Jalan Menara Gading 1, Taman Connaught, Cheras, 56000 Kuala Lumpur, Malaysia

## Abstract

K-*ras* is an oncogenic GTPase responsible for at least 15–25% of all non-small cell lung cancer cases worldwide. Lung cancer of both types is increasing with an alarming rate due to smoking habits in Malaysia among men and women. Natural products always offer alternate treatment therapies that are safe and effective. *Typhonium flagelliforme* or Keladi Tikus is a local plant known to possess anticancer properties. The whole extract is considered more potent than individual constituents. Since K-*ras* is the key protein in lung cancer, our aim was to identify the constituents of the plant that could target the mutated K-*ras*. Using docking strategies, reported potentially active compounds of *Typhonium flagelliforme* were docked into the allosteric surface pockets and switch regions of the K-*ras* protein to identify possible inhibitors. The selected ligands were found to have a high binding affinity for the switch II and the interphase region of the *ras*-SOS binding surface.

## 1. Introduction

Cancer is a major health problem in Malaysia with a total of 2,048 cases registered with National Cancer Registry (NCR) in 2006 [1]. The disease is now the third leading cause of premature deaths in our country. Lung cancer is among the top five cancers affecting both male and female in Malaysia at 9.4 percent in peninsula Malaysia with 2100 Malaysians diagnosed every year [2, 3]. Smoking related diseases are increasing in Malaysia especially lung cancer [3, 4]. A recent study conducted by Liam et al. (2013) highlighted adenocarcinomas as the most frequent types of cancer among Malaysian men and women, smoker, and nonsmokers. With an incidence rate of 109.8 cases of cancer per 100,000, it is imperative to find treatments that are safe and effective [5].

Lung cancer can be divided into two major classes based on its biology, therapy, and prognosis, namely, non-small cell lung cancer (NSCLC) and small cell lung cancer (SCLC). NSCLC accounts for 75% to 80% of all lung cancer incidents while small cell lung cancer accounts for 15% to 25% of all lung cancer [6].

The* ras* pathway is an important signaling pathway that allows cell proliferation in response to stimulation of the epidermal growth factor receptor [7, 8]. These signals affect the production and regulation of other key proteins involved in cell proliferation. Studies have reported that K-*ras* mutation occurring in NSCLC varies between 16% and 40% [8]. K-*ras, *a GTPase, also known as V-Ki-ras-2 (Kirsten rat sarcoma viral oncogene) is a protein that in human is encoded by the K-Ras gene [9]. The normal protein is an essential part of the* ras* signaling pathway acting as a molecular switch. In the “off” state, it is bound to the guanine diphosphate nucleotide (GDP). It is turned on via the growth factor stimuli. The guanine nucleotide exchange factor, also known as the son of sevenless (SOS) protein, and the growth factor receptor-bound protein 2 (Grb-2) together enable the K-*ras* to become activated by exchanging the GDP molecule for the more active guanine triphosphate nucleotide (GTP). Once turned “on,” it recruits and activates downstream proteins necessary for the propagation of growth factor and other receptor signals. It possesses an intrinsic weak enzymatic activity which is enhanced by interaction with the GTPase activating protein (GAP) leading to cleavage of the terminal phosphate of the nucleotide guanine triphosphate (GTP) converting it into the guanine diphosphate (GDP). Upon conversion of GTP to GDP, K-*ras* is switched “off.” Despite being a weak GTPase, K-ras possesses two very important features involved in its switching “on” and “off.” Called switches because of their ability to change the conformation of the protein in the active and inactive state, the regions are involved in interacting with the nucleotides. The guanine nucleotide pocket of the K-*ras* is highly conserved and is lined with residues 11–16 [10, 11]. Because of the specific interactions of amino acid residues of this region with the GTP, mutations at the 12 and 13 amino acid positions in the enzyme lead to permanent cell proliferation because it cannot be hydrolysed and hence, the* ras* signaling function is unable to be turned “off” [12–14]. The mutated K-ras (mut-Kras) is an interesting drug target of several studies [15–18]. The major reason being that it provides fast resistance to the available drug therapy. Several EGFR, MEK inhibitors have been tried in single and combination. However, drug resistance develops quickly [17, 19–21].

Medicinal plants with anti-cancer effects are commonly used as alternative medicine because of their safety and toxicity profiles. Several herbal medicines have been studied for finding effective treatment of lung cancer. Only few studies on the use of Malaysian medicinal plants as treatment options have been reported [4, 21, 22].* Typhonium flagelliforme* (Keladi tikus) is one such plant that is found locally in Malaysia that has been studied for its inhibition of proliferation in human lung cancer cell line. Its active ingredients including phytol and its derivative, hexadecanoic acid, 1-hexadecene, and pheophorbide related compoundshave shown some promising results as anticancer when whole extracts have been used. Lai et al. reported that they could not find single constituents as effective when compared to the extract [22, 23].

Computer studies have recently provided insights into the mechanics of K-ras protein [24–26]. Researchers have offered in depth study of the various mutations and the effect they have on the “on-off” states of the protein. With sophisticated software being available to researchers, they have recently reported direct inhibition of the protein as therapeutic target. Maurer et al. (2012) have carried out an in-depth study of the allosteric binding pockets on the protein that maybe targeted in the “off” state of the mut-K-ras [24].

In this study we explored the inhibitory effects of the some of the reported potent constituents of* T. flagelliforme* on the lung cancer cell lines using docking studies with Autodock Vina [27]. We used the reported structures of the active ingredients and docked them into reported allosteric binding sites [25, 28] on the mut-K-ras to determine the probable binding sites of the constituents. We also tried to relate the experimental results obtained by Lai et al. [22, 23] to our computational observations to gain meaningful insights into the use of the proposed plant constituents as probable inhibitors.

## 2. Materials and Methods

The three dimensional structures of the G12D mutated K-ras structure inhibitor bound to SOS pocket (PDB ID: 4DST), and two G12C mutated K-ras structure bound to allosteric sites (PDB ID: 4LUC and 4LYF). Autodock Vina 4.2 [27] was used to dock all ligands to the K-*ras* protein. Before that, we used Autodock tools downloaded from The Scripps Research Institute to prepare the ligand and protein file [29, 30]. All water molecules were removed and Kollman charges added as described in the Autodock Vina 4.2 manual [31, 32]. The grid box dimensions were obtained from the grid box widget by keeping the bound ligand sites as box centers. Control studies were performed with all ligand bound in the crystal structures before docking with test ligands from* T. flagelliforme*. Pheophorbide a and two related epimers were drawn in ChemSketch [33] based on reported structure by Lai et al. [22] while hexadecene and hexadecanoic acid structure were obtained from Pubchem. The 2D structures of the active constituents are tabulated in Table 1.

## 3. Results and Discussions 

The results of highest binding affinity of the five active constituents for the GTP bound K-*ras* as well as the mutated protein structures are tabulated in Table 2.

*T. flagelliforme* is a local Malaysian plant with anticancer activity when taken as a fresh juice prepared from freshly crushed plant. Researchers have reported its activity in lung cancer as a whole extract in dichloromethane [22, 23]. Lai et al. (2010) reported that the extract contained at least 11 chemical compounds of hydrophobic character [22]. The most predominant compounds were pherophorbide a, pheophorbide a′, pyropheophorbide a, methyl pyropheophorbide a, hexadecanoic acid, oleic acid, linoleic acid, linolenic acid, campesterol, stigmasterol, and *β*-sitosterol. The* in vitro* studies conducted by the group on the NCI-H23 lung cancer cell line had concluded that individual compound isolates had failed to show a significant anti-cancer behavior. However, the whole extracts were found to have an IC_50_ of 2.7 *μ*g/mL suggesting that components had a synergistic effect on antiproliferation of cancerous cells. They also suggested that the constituents showed increased activity upon exposure to light. The authors had also earlier reported an active whole extract of the plant comprising of hexadecanoic acid, 1-hexadecene, phytol, and a phytol derivative with an IC_50_ of 7.5 *μ*g/mL against NCI-H23 cell lines [23].

Mohan et al. (2010) conducted their study on the leukemic cells and showed the selectivity of the dichloromethane fractions for the cancerous cells. However, they did not indicate the effect of any particular constituent on the cancer cells [34].

In our study we focused on determining whether the proposed constituents by Lai et al. (2008, 2010) were able to target the K-*ras* protein either directly or allosterically. For direct inhibition we docked the selected ligands at or near the SOS binding pocket using the PDB structure 4DST [24]. The target binding site proposed by the authors is near the* ras* switches I and II and binding to this area was indicated as interfering with* ras*-SOS binding surface [24]. This finding is important because the* Ras*-SOS complex is essential for activation of the K-*ras* since SOS initiates the GTP exchange process to the protein. According to the resolved structure of the* ras*-SOS complex (PDB ID: 1BKD) [35], the CDC25 binding region is tightly bound to the Switch II of* ras* and causes the disruption of the GDP bound structure [35]. Tyr 64 of the* ras* appears to be the anchoring residue for the* Ras*-SOS complex. Hence, the inhibitors should be designed to target the switch regions or the binding surface between the protein-protein complex. Where small molecules can modulate the switch regions of the* ras* due to space confinement, larger molecules can target the accessible surface areas between the protein-protein complex.

**Figure 1:**
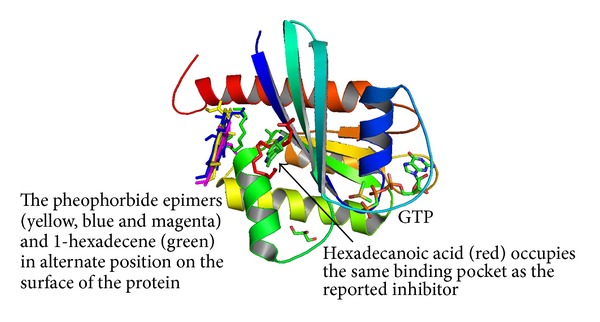
Docked poses of* flagelliforme* constituents to GTP bound K-*ras.*

Our semiflexible docking experiment on the K-ras molecule with control ligand 4,6-dichloro-2-methyl-3-aminoethyl-indole (4DST) [24] had a binding affinity of −5.4 Kcal/mol. Among the test ligands the highest binding affinities were shown by the pheophorbide epimers. The observed docked poses, given in Figure 1, showed that the epimers were not near the binding site of the control ligand.

The test ligand that showed affinity for the same binding site as the control molecule was hexadecanoic acid with binding affinity −4.1 Kcal/mol. It appeared to overlap the control molecule at the same position. The authors suggested that the binding pocket involves residues Lys5, Leu6, Val7, Ile55, Leu56, and Thr74 [24]. Our results showed mostly the same binding site except that Thr 74 was placed a bit further from the ligands but within 5 Å. Our results showed the binding pocket residues to be Lys5, Leu6, Val7, Glu37, Ser39, Arg40, Asp54, Ile55, Leu56, Gln70, Tyr71, Met72, Thr74, and Gly75. They further elaborated that the binding of the indole derivative expanded the pocket to accommodate the ligand. A recent report by Grant et al. (2011) also established this region as one of the allosteric binding pockets important in finding inhibitors for K-ras [25]. Hexadecanoic acid is a long chained hydrocarbon that folds into a U-shape when docking into the pocket. This folding makes the molecule snugly fit into the pocket. From this result we can assume that when the cancer cells were incubated with the* T. flagelliforme* extract [23], the hexadecanoic acid could possibly target the K-ras at this surface pocket.

The other ligands presented another interesting position. The pheophorbide epimers docked strongly into a depression on the surface of the K-ras that is also the surface for interaction with the SOS protein [35] The residues involved that formed the binding pocket for the epimers and 1-hexadecene were Arg73, Thr74, Gly75, Glu76, and Gly77. The strong binding affinity between the protein and the epimers was the result of the hydrogen bonding between the ligands and Arg73 and Gly75.

For another docking experiment we used two of the PDB structures, 4LUC and 4LYF, reported by Ostrem et al. (2013) since it presented two different ligands, a sulphonamide and a vinyl sulphonamide that caused changes in the switch II region to accommodate the ligand [28]. The researchers focused on finding inhibitors that could bind to mutated GDP bound K-ras and change its structure such that it would not be able to exchange the GDP molecule for the GTP to be activated.

The authors showed that their test ligands targeted the switch II region that falls in the loop region between the central *β*-sheet, α2 and α3 helices of the* ras*. The pocket had been earlier reported by Taveras et al. [36]. We used the same binding pocket lined with residues Val7, Val9, Gly10, Ala11, Thr58, Ala59, Gly60, Gln61, Glu62, Glu63, Arg68, Tyr71, and Met72. Our results revealed that the pheophorbide epimers had the strongest affinity. However, the docked poses presented an entirely different picture.

**Figure 2:**
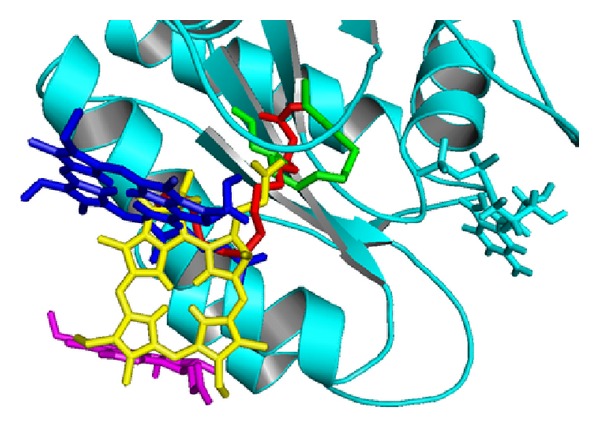
Docked poses of the* flagelliforme* constituents to the binding pocket of K-ras (PDB ID 4LUC). The ligands are hexadecanoic acid (red), pheophorbide a (yellow), phephorbide a′ (blue pyropheophorbide a, and magenta), and 1-hexadecene (green)).

In case of the structure PDB ID: 4LUC, pheophorbide a could insert its side chain (–CH_2_–CH_2_–COOH) into the pocket (Figure 2). The same side chain of pheophorbide a′ did not penetrate much. Pyropheophorbide a occupied position lined by residues Phe90, Glu91, His94, His95, Gln129, Asp132, Leu133, and Ser136. Being bulky molecules they could not penetrate inside the pocket, however could make a hydrogen bond with Glu63, His95 and Tyr96 that contributed to the binding affinity of −7.0 for pheophorbide a, 6.8 for pheophorbide a′ and −7.3 Kcal/mol for pyropheophorbide a. Hexadecanoic acid and hexadecene could easily slide into the pocket and make polar contact with Arg68. This was perhaps due to the long hydrophobic chain despite the low binding affinity. The pheophorbide a and a′ showed that they occupied the binding region that included residues 61–64 and 68 on K-*ras *with pheophorbide a interacting with Glu63. As already pointed out [35] these residue are involved in the contact surface between the* Ras*-SOS proteins. Hence if pheophorbide is able to bind to these residues, it could prevent the interaction of the two proteins in a pronounced manner.

The other K-ras structure (PDB ID: 4LYF) has a vinylsulphonamide covalently bound to the K-*ras* in the same pocket area. The authors indicated that when this compound binds to the pocket, it modifies the switch II position and completely disorders switch I [25].

**Figure 3:**
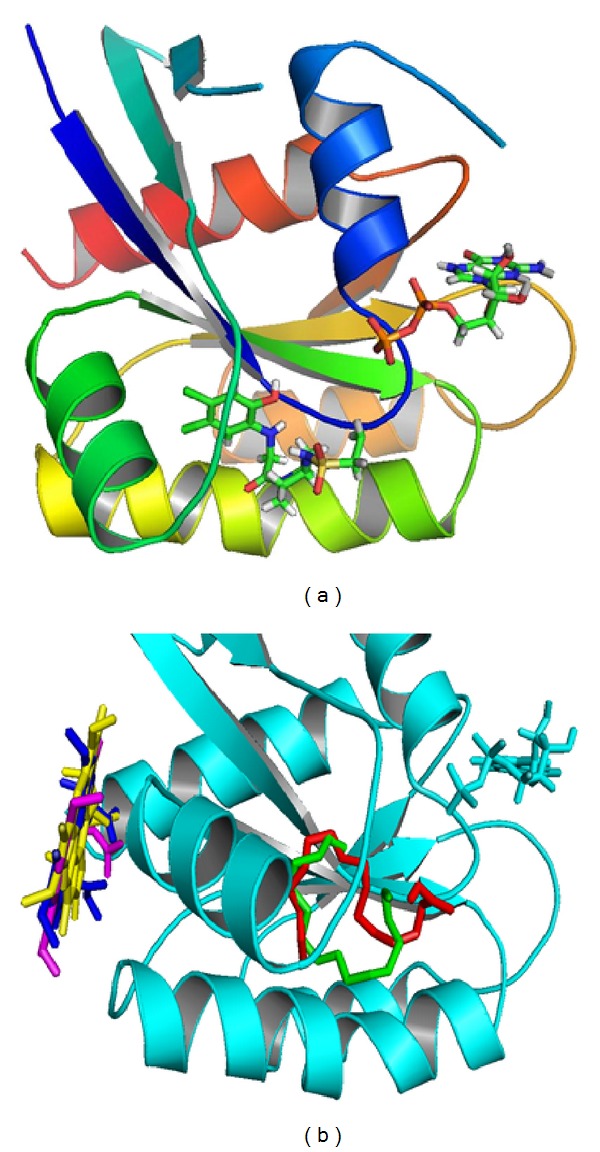
Docked poses of the* flagelliforme* constituents to the binding pocket. K-ras PDB ID 4LYF. (a) Control ligand vinylsulphonamide docked in the switch II region. (b) Test ligands pheophorbide a (yellow), pheophorbide a′ (blue), and pyropheophorbide a (magenta) bound at the SOS-interaction site while 1-hexadecene (green) and hexadecanoic acid (red) are bound in the switch II region.

Our docking experiment given in Figure 3 on this expanded pocket allowed the 1-hexadecene and hexadecanoic acid to go deep into the pocket and occupy the same binding pocket as the inhibitor and formed hydrophobic interactions inside their binding pocket. As had been pointed out by the authors, the disorderliness of the switch regions caused by the ligands could lead to SOS protein not being able to bind efficiently to the* ras* protein, leading to inhibition of the exchange of nucleotides and ultimate inactivity of the protein [25]. However pheophorbides targeted the binding region comprised of Lys5, Arg73, Thr74, Gly75, Glu76, Val103, Lys104, Asp105, and Ser106 which according to Boriack-Sjodin et al. (1998) is part of the site of interaction between* ras-SOS *[35]. Pheophorbide a exhibited the highest affinity of −6.7 Kcal/mol, while pyropheophorbide a molecule with binding affinity 6.5 Kcal/mol formed a hydrogen bond with Arg73. Because of their big size and span of the ring, we can hypothise that these compounds cover the area of interaction between K-*ras* and SOS and, hence, may be important direct inhibitors of the K-*ras* at the* ras*-SOS interacting region.

Taking these results together, we can say that K-*ras* could be a good target for the pheophorbides of the* flagelliforme*. They have two hotspots on the protein. One is the region of surface contact between the SOS protein and the K-*ras* and the other could be the switch two region. We carried out experiments in both the activated state and inactivated state and in both states. Our results showed that pheophorbides naturally targeted the SOS interacting residues preferable in the activated (4DST) and inactivated state (4LUC, 4LYF). The hexadecanoic acid and 1-hexadecene looked for hydrophobic pockets that it could slide into and their preferable hotspot was switch II.

## 4. Conclusion

Hence, we can conclude that* T. flagelliforme* constituents could target several allosteric sites on the K-*ras*. Since this protein is the most important signaling molecule in cancer cells inhibiting this protein would induce apoptosis of lung cancer cells. Combining our results with experimental evidence from Lai et al. (2008, 2010) where whole extracts are more potent than individual constituents, we can hypothesize that K-*ras *could have been the probable target of pheophorbides and other constituents. Pheophorbides bind to the SOS binding spot on K-*ras *and could possibly prevent a strong interaction between the nucleotide exchange protein SOS and K-*ras, *while 1-hexadecane and hexadecanoic acid bind to switch II region of the K-*ras*. Both events combined would eventually inhibit the growth signals in the cancerous cells. Further studies are required to conclusively indicate K-*ras* at the target for the pheophorbides and other constituents.

## Figures and Tables

**Table 1:** Two dimensional structures of the constituents used in the study.

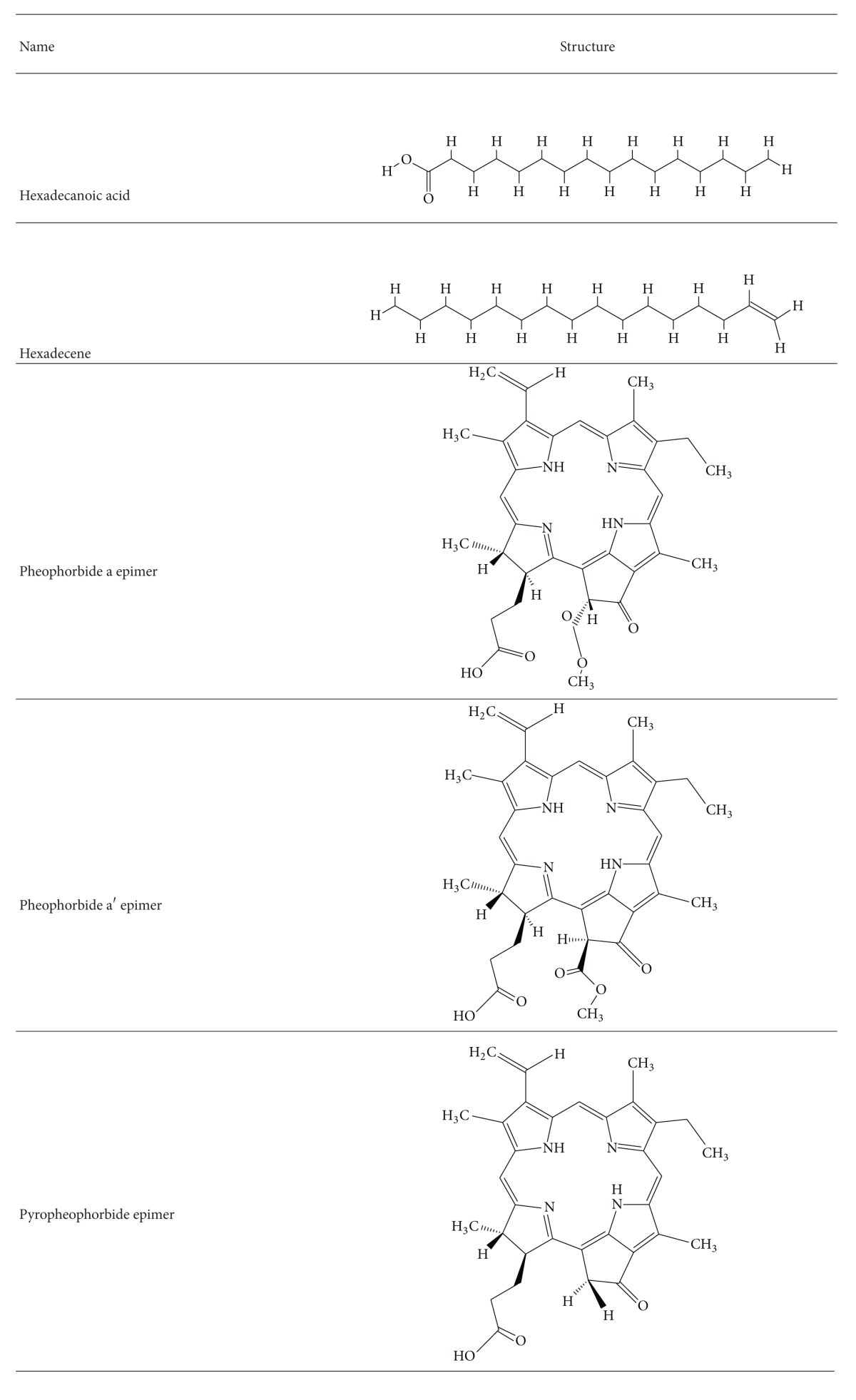

**Table 2:** Binding affinity values obtained for the control and test ligands.

Protein	Binding affinity Kcal/mol					
	Control	Pheophorbide A	Pheophorbide A′	Pyropheophorbide A	1-Hexadecene	Hexadecanoic acid
G12D mutated K-ras GDP SOS binding pocket (4DST)	−5.4	−7.2	−7.5	−7.1	−4.0	−4.1

G12C mutated K-ras GDP allosteric binding pocket (4LUC)	−8.0	−7.0	−6.8	−7.3	−4.8	−4.6

G12C mutated K-ras GDP allosteric binding pocket (4LYF)	−6.7	−6.7	−6.6	−6.5	−4.5	−5.0
